# Telehealth Use and Legal Considerations in Drug Health Services During Pandemics: Systematic Scoping Review

**DOI:** 10.2196/46394

**Published:** 2024-11-12

**Authors:** Meryem Jefferies, Robert Graham, Marguerite Tracy, Scott Read, Mohammed Eslam, Mark W Douglas, Jacob George

**Affiliations:** 1 Drug Health, Western Sydney Local Health District North Parramatta, NSW Australia; 2 Storr Liver Centre, The Westmead Institute for Medical Research The University of Sydney at Westmead Hospital Westmead, NSW Australia; 3 General Practice Clinical School, Faculty of Medicine and Health, The University of Sydney Sydney NSW Australia; 4 Blacktown/Mt Druitt Clinical School and Research Centre, School of Medicine, Western Sydney University Blacktown, NSW Australia; 5 Blacktown Hospital, Western Sydney Local Health District Blacktown, NSW Australia; 6 Centre for Infectious Diseases and Microbiology, Sydney Infectious Diseases Institute, The University of Sydney at Westmead Hospital Westmead Australia

**Keywords:** telehealth use, emergency department admission, substance use disorder, drug addiction, consent, privacy, professional indemnity, confidentiality, data security

## Abstract

**Background:**

The COVID-19 pandemic impacted patients with substance use disorder (SUD) more than the general population and resulted in substantially increased emergency department admissions. Routine care of patients attending drug health services during the pandemic transitioned, with telehealth being important in delivering appropriate care. However, telehealth introduces unique risks such as privacy, confidentiality, and data safety. Providing health care through telehealth may fail if the legal impacts are not fully identified and acted on by health professionals. It also poses unintended risks for patients and can result in ineffectiveness, damages, medical negligence, and detracts from the best intentions of governments and health professionals. Understanding the legal framework ensures that medical professionals operate health care through telehealth within the law. Providing health care successfully through telehealth depends on the balance between innovation and legal compliance. By considering these aspects, clinicians and practitioners can provide effective and safe telehealth services during pandemics or any other natural disaster.

**Objective:**

We aimed to explore the legal impact of autonomy consent, confidentiality, privacy, data security, professional indemnity, and liability when delivering telehealth to patients with SUD. The scoping review also aimed to provide legal, ethical, and clinical considerations to minimize legal risks with using telehealth in drug health service outpatient settings.

**Methods:**

We performed a scoping review to provide an overview of existing research, statutes, and case laws for the incorporation of clinical, ethical, and legal considerations into telehealth use. Six databases for medical and 6 databases for legal publications were searched, as well as Australian national and selected international regulatory standards. Medical articles published up to June 2022 were included in this review. Our search yielded 1436 publications, 614 abstracts were reviewed, and 80 published studies met the inclusion criteria from 614 legal and medical search results. Current regulations related to technology use in drug health services, relevant cases, and international regulatory standards are discussed.

**Results:**

In total, 43 legal documents including 15 statutes, 4 case laws, and 37 medical publications were reviewed. The themes arising from the literature were consent and autonomy (20/80, 25%), confidentiality (8/80, 10%), privacy (8/80, 10%), data security (7/80, 9%), and professional indemnity issues (3/80, 4%) in telehealth use. Further, 24 studies identified legal issues associated with telehealth use in patients with SUD.

**Conclusions:**

Our review identified potential legal issues associated with telehealth use in patients with SUD. Several legal and medical research articles provide frameworks, codes of conduct, or suggestions for clinicians to consider, but there was little discussion or evidence of how legal considerations are being applied when providing telehealth consultations at drug health services. Clinicians should be aware of the medicolegal implications when providing health care via telehealth at drug health services.

## Introduction

A recent study showed a substantial increase in emergency department presentations for patients with substance use disorder (SUD) during the COVID-19 pandemic [1]. This population required innovative solutions [2] to provide access to timely health care [1] including the use of telehealth. New technologies however raise new legal, security, and ethical considerations [3]. Due to innovations developing faster [3], implementing an appropriate technology that ensures patient safety while delivering the best possible care to patients with SUDs is important [2]. The time frame for creating appropriate clinical frameworks within the current regulatory structure however is not adequately addressed [4].

There is currently no published study analyzing the use of telehealth and the related issues that arise in the clinical context of drug health. This review outlines using telehealth to deliver routine clinical care to patients attending drug health services in emergencies, identifies specific issues that arise from the use of such platforms with current laws, and describes the elements to be considered by clinicians when using telehealth at drug health services.

The Australian Government responded to the COVID-19 pandemic [5] by legislating the COVID-19 Legislation Amendment (Emergency Measures) Act 2020 [6] to regulate the public health emergency [7]. Likewise, New South Wales (NSW) Health implemented interim actions for surveillance, infection control, laboratory testing, and contact management for COVID-19 [8]. In the context of a pandemic, people attending drug health services have an increased risk of transmission of infectious agents compared to patients with other health conditions. People requiring drug health services are often required to attend clinics daily, increasing the risk of transmission of respiratory viruses. Several studies on the impact of the COVID-19 pandemic on people with SUD have reported higher rates of depression, anxiety, irritability, and posttraumatic stress [9]. To meet the challenges, digital health services were increasingly used in drug health services, including *My Health Record* [10] and telehealth [11].

Using telehealth will continue to provide health care in drug health services. However, there remains a need for a legally regulated model of care for the use of telehealth to deliver best practices for patients with SUD. In this paper, we addressed potential legal issues about autonomy and consent, confidentiality, privacy, data security, professional indemnity, and liability when using telehealth in drug health services.

## Methods

### Study Design

As this review involved legal and medical considerations, a systematic scoping review is the most suitable methodology. We conducted the scoping review by following the PRISMA-ScR (Preferred Reporting Items for Systematic Reviews and Meta-Analyses extension for Scoping Reviews) guidelines. This checklist is provided in Multimedia Appendix 1 [12,13].

### Review Context

This review context is of what is known from the legal and medical literature about the use of telehealth, current regulations related to telehealth use in drug health services, relevant cases, and international regulatory models.

### Inclusion Criteria

Inclusion criteria were established before performing the search as shown in Table 1. We performed the search up to June 2022 and used relevance, article type, publication sources, and subject matter for including and excluding publications. As relevance, those related to telehealth use in patients with SUD, autonomy and consent, confidentiality, privacy, data security, and professional indemnity and liability were included. As article type, original studies, reviews and editorials, viewpoints, guidelines, letters to editors and commentaries, and relevant legislation and case laws were included. As publication sources, published papers in a peer-reviewed platform or an institutional report were included. As subject matter, those related to regulations and legal considerations when using telehealth in drug health services, autonomy and consent, confidentiality, privacy, data security, and professional indemnity and liability were included. We excluded any publication that was not related to telehealth use in drug health services. Legal articles that were not published in full or accessible, preprints, or unavailable full sources were also excluded.

**Table 1:** Inclusion and exclusion criteria.

Type of criteria	Included	Excluded
Relevance	Telehealth, SUD^a^, autonomy and consent, confidentiality, privacy, data security, professional indemnity, and liability	Those irrelevant to telehealth use or did not contain a telehealth component in patients with SUD
Article type	Original studies, reviews, editorials, viewpoints, guidelines, letters to editors, commentaries, relevant legislation, and case laws	Preprints
Publication sources	Published in a peer-reviewed platform or institutional report	Unavailable full texts or full names of sources
Subject matter	Regulations and legal considerations, telehealth, drug health service, autonomy and consent, confidentiality, privacy, data security, professional indemnity, and liability	Those related to telehealth modelling, and studies that used technology only for a better understanding of disease dynamics with no help from health professionals

^a^SUD: substance use disorder.

### Search Strategy

This review was performed with two search strategies—a medical and a legal search.

### Medical Search

Research on telehealth use in patients with SUDs was performed using the key databases MEDLINE, Embase, and CINAHL. Databases including the Science Citation Index were expanded by using database-specific controlled vocabulary (where available) and general free-text terms. Other relevant websites were explored including those of the Global Health Library, WHO, United States Food and Drug Administration, European Medicines Agency, and Australian Government Department of Health. Other search engines such as Google Scholar, ResearchGate, and Science Direct were explored using the search terms. Keywords searched include telehealth use, emergency department admission, SUD, drug addiction, consenting, privacy, confidentiality, and data safety. Credibility and quality of sources were evaluated by reviewing the reputation and authority of the sources. The medical search was performed on titles and abstracts. All publications stating the role of telehealth use in drug health services during the COVID-19 emergency, autonomy and consent, and data security were included in the analysis. Duplicate publications, review articles, opinion articles, and letters were excluded. Those publications which did not provide the principal data or were articles with incomplete data were excluded.

### Legal Search

The legal search was undertaken using Australian Case Law databases including Westlaw AU, Lexis Advance Pacific, AustLII, CCH Intelliconnect, and CaseLaw NSW. The relevant legislation in Australia, the United States, the United Kingdom, Canada, and New Zealand were reviewed. The key terms including autonomy consent, confidentiality, privacy, data security, professional indemnity, liability, Privacy Act, and legal basis for telehealth were used.

The search was conducted on titles, abstracts, and a summary of case laws. An elementary search on June 1, 2021, identified a range of evidence on the role of technology in drug health services during the COVID-19 pandemic. A combination of keywords: COVID-19, emergency department admission, telehealth in health care, telehealth in drug health clinic, eHealth, e-consenting, telemedicine, SUD, autonomy, and consent, data security, privacy, and confidentiality were used. Relevant publications and legal authorities were selected for further review. A review of authoritative judgments regarding consent and patient record privacy was included.

### Article Selection and Data Extraction

All studies stating the role of telehealth use in drug health services during COVID-19, autonomy and consent, and data security were included in the analysis. Duplicate publications and papers not providing the principal data or articles with incomplete data were excluded. Case authorities reviewed are of superior courts and apply to the current context of drug health in Australia.

We retrieved 300 records from our medical search and 314 records from our legal search. Then 496 records were screened for the relevance of title and abstract, excluding 260 of the records. Additionally, 156 were further excluded as they were not directly relevant to the context of this study. Finally, 80 studies (43 legal and 37 medical studies) were included. The bibliography of all selected articles was reviewed for relevance.

## Results

### Overview

**Figure 1:**
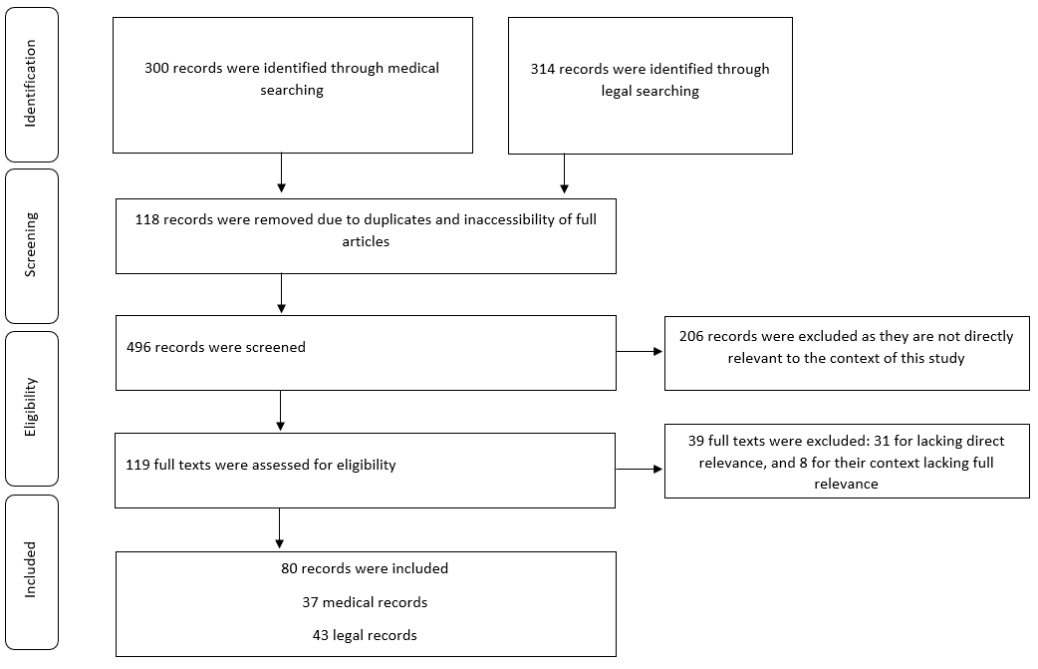
PRISMA (Preferred Reporting Items for Systematic Reviews and Meta-Analyses) flow diagram of the review process.

A summary of medical and legal studies used in this manuscript is outlined in the PRISMA (Preferred Reporting Items for Systematic Reviews and Meta-Analyses) flow diagram (Figure 1). Medical issues arising from the published literature included those at the health system level—cost-effectiveness, access, early intervention [9] to prevent use of emergency services [1]; local level practice issues—cost of technology [2], ensuring data safety and privacy [14]; clinician level—conducting a thorough clinical assessment via telehealth, medicolegal indemnity cover [15]; and at the patient level—increased access to care, potential for misdiagnosis, data security and confidentiality [14], inequitable access to technology and connectivity [16].

From the records assessed, five domains should be considered by health professionals when providing health care in the drug health context; these include autonomy and consent, confidentiality, privacy, data security, and professional indemnity and liability.

The reviewed literature offered several positive aspects stemming from the use of technology platforms to enhance health care delivery in drug health. These include cost-effectiveness, the ability to provide health care to remote and regional areas, the potential to reduce infection transmission, and a reduction in emergency department admissions.

While several positive aspects of the use of telehealth in drug health care were identified, the literature also identified numerous issues: patient privacy, data privacy [17], the risk of cybercrime [18], data security, lower efficacy for clinical examination, health professional licensure, reimbursement, credentialing, malpractice [19], and high risk of misdiagnosis due to the inability to conduct a complete physical examination [20]. In one report, 63% of total misdiagnoses in health care were the result of failing to perform a physical examination [21]. There are numerous local reports on security breaches that result in a patient’s medical information being accessed by unauthorized third parties [22,23] A further issue is legal uncertainty regarding professional indemnity for clinicians during remote consultations [24].

### Legal Considerations in the Use of Telehealth in Drug Health Clinical Settings

#### Autonomy and Consent

This review identified several legal issues regarding obtaining consent from patients with SUD through telehealth. Philosophers and ethicists [21] have proposed arguments to suggest that people with SUD may be unable to provide informed consent to participate in treatment programs [25]. Their reasoning included the inability to say “no” to the offer of heroin [26]. Other research however suggests that addiction does not significantly impair autonomy. In NSW, patient consent to treatment is mandatory [27,28] except for involuntary treatment in cases of severe substance dependence under the Drug and Alcohol Treatment Act 2007 (NSW) [29]. The legislation provides that patient consent to treatment is not required under 3 limited circumstances. First, when an emergency arises and the patient lacks capacity and treatment wishes are unknown [30], when capacity is deemed by law to not exist—such as when patients are legally “incompetent” due to mental health or disability (in such instances legislation provides for alternate means of obtaining consent), and where the law overrides a patient’s right to provide consent [31].

Where treatment is given without consent, or it exceeds the terms of the consent, the treatment will constitute a tortious battery and may also be the subject of complaint and disciplinary sanction. This is the case even if the treatment is successful or therapeutic. Where treatment is given and the consent is premised on incorrect or inadequate information, a patient has a cause of action in negligence if they endure damage or loss.

In most treatment contexts, obtaining informed consent is a necessary precondition of treatment. Without consent, a medical practitioner may face disciplinary allegations of professional malpractice or be sued for battery or negligence if detriment or harm is endured. This is despite the fact the treatment may be given in a manner that benefits the patient.

Obtaining patient consent via telehealth in a drug health setting can be problematic. Currently, there is no legal authoritative case regarding breach of consent due to telehealth use. However, the following cases are reflective of patient consent-related issues which may arise in drug health clinics.

The accepted legal test at common law for patient consent and autonomy is established in Rogers vs Whittaker [32,33] and the test has also been adopted in Canada and the United Kingdom [34]. This legal principle potentially applies to telehealth use if a health care professional fails to obtain informed consent and an informed decision from the patient regarding the risks of particular procedures and if consultation is not performed in person. The case is considered a precedent in many countries including the United Kingdom, the United States, New Zealand, and Canada [35]. Courts have no difficulty in assessing the presence and efficacy of consent simply because information and treatment are being provided by using technology.

There are challenges to providing health care via telehealth which present new variables and challenges that are not present in face-to-face consultations. The risk of misreading nonverbal cues in telehealth, the inability of the patient to access relevant information (due to their own limitations or the lack of availability of this information), the digital literacy of the patient and the effect of influence by other persons who may be unknowingly present when telehealth is conducted are all variables that have the potential to impact on the exchange of information, and therefore on the practitioner’s ability to determine informed consent.

In Malette vs Shulman [36], the issue of lack of consent and autonomy was considered. The plaintiff (Malette) was seriously injured in a car crash and was rushed to a hospital where she was found to carry a card that identified she was of the Jehovah’s Witness faith, and expressly requested that no blood transfusions be given under any circumstances. However, Dr Shulman authorized a life-saving blood transfusion despite knowing that the procedure was contrary to the patient’s consent. The patient sued. The Supreme Court of Ontario concluded that the doctor had violated the patient’s rights over her body by acting against her intentions without consent [37] and this constituted a battery. Battery is a legal concept that occurs when a person touches or applies force to another person’s body in an unauthorized and offensive way. In Malette vs Shulman, the blood transfusions without Georgette’s consent raised the question of whether it established a battery. The rules of self-determination and individual autonomy play an essential role. Georgette’s right to reject treatment, even if it might have the purpose of lifesaving, constitutes her autonomy. Dr Shulman’s action against Georgette’s consent in favor of saving her life constituted a legal and ethical tension. The trial judge decided in favor of Georgette, highlighting that the Jehovah’s Witness card validly limited Dr Shulman’s right to treat her. Dr Shulman’s actions constituted a battery as there were no grounds to ignore Georgette’s obvious rejection of blood transfusions. Malette vs Shulman provides the nuanced balance between respecting patient autonomy and ensuring medical help. It highlights the legal obligation to obtain informed consent, even in lifesaving or any other emergencies. The case can be considered as a precedent when issues of consent and patient autonomy arise in telehealth use. The case is recognized in multiple countries including Australia, the United States, the United Kingdom, and New Zealand in addition to Canada [35].

The relevant legislation in NSW regarding consent to treatment are the Children and Young Persons (Care and Protection) Act 1998 [29,38,39], Guardianship Act 1987 [40,41], Guardianship Regulation 2016 [42,43], and Minor (Property and Contracts) Act 1970 [42,44], and Drug and Alcohol Treatment Act 2007 (NSW). Obtaining and ensuring informed consent is an essential domain for telehealth consultations of patients for drug health and providing health care without consent can lead to risk of malpractice and medical negligence [45]. Consent must be provided freely [46] and the use of telehealth should acknowledge and account for the limitations and risks of telehealth services when compared to face-to-face treatment. Obtaining consent through telehealth also faces legal and regulatory barriers such as large variations in rules, regulations, and guidelines for practice. Such variation contributes to the confusion of providers engaged in the practice of telehealth [47]. The Medical Board of Australia provides guidelines on technology-based patient consultations and their medicolegal implications [48].

#### Confidentiality

Patient confidentiality is at risk of being breached when using telehealth. Information related to health is always sensitive and drug health clients always prefer that their medical diagnoses and treatment be handled with a high level of confidentiality [49]. Moreover, health care providers need to inform the patient that they have a right to access information about themselves, to approve the information to be used by other health care providers, and in some cases for deidentified information to be used in clinical research [49].

Skene [15] defined confidentiality from three viewpoints. The first is an ethical perspective, as maintaining confidentiality about a medical record respects the autonomy of the patient [45]. The second is the medical perspective. If a patient knows that what they discuss with their doctor is confidential, they are more likely to be honest about what they disclose, which is beneficial for their treatment [45]. The third is a public policy perspective, that is, it is in society’s interest that patients are provided with the best possible treatment through telehealth [15].

Medical records of patients with substance use dependency and mental health issues have strong protection in the United States. In Jaffee vs Redmond [33]*,* communications between psychotherapists and their patients were found to be privileged under the Federal Rules of Evidence [50]. This judgment extended the privilege of patient records to psychotherapists, with the court noting that confidentiality played an important part in effective patient treatment. The case law is authoritative in other states because of the superiority of the court making the judgment [50]. The court concluded that the privilege is necessary because “the mental wellbeing of our citizenry, no less than its physical health, is a public good of transcendent importance” and “the mere possibility of disclosure may harm establishment of the confidential relationship necessary for successful treatment” [50].

In summary, health professionals must consider confidentiality when providing health care through telehealth by taking reasonable steps such as ensuring the use of telehealth software that prevents screen recording [48]. However, confidentiality can be still breached such as by recording the consultation using another device [48]. Jaffee vs Redmond is one of the key cases for legal and ethical consideration when using telehealth regarding patient confidentiality and is recognized around the world [51].

#### Privacy

Patient privacy can be compromised in the following ways: first, patients may need to access the internet from public locations such as libraries due to limited connectivity. This situation can compromise privacy, as patients may be using shared devices or networks, potentially exposing sensitive health information. Living in a remote area without an internet connection is not a privacy issue, however, lack of internet connection and the act of using the internet from public places can impact privacy [48]. Second, health professionals might have less ability to perform a controlled examination through telehealth. This can lead to a lack of control over the collection, use, and sharing of private information. Unusually, people may be around the consultant and the patient might not want to overhear what was said or sometimes people are not comfortable discussing health issues from within their home due to a lack of privacy, and an unsafe environment to discuss concerns [52]. Patients for drug health are a vulnerable cohort and when their medical information is disclosed against their will, they frequently feel ashamed, helpless, and stigmatized [53]. Successful treatment may become difficult in such circumstances and the relationship of trust with the doctor may be permanently harmed. Actual or threatened disclosure can be particularly traumatic for the drug health of patients as they need supportive, reliable, and trusting relationships with health care providers.

The following cases are also relevant in Australia. In R vs Osolin [54], the importance of patient-centric privacy was emphasized by the court and the case is recognized by multiple jurisdictions around the world [55]. In this case, disclosure of sexual assault in medical records that were then produced under subpoena was considered by the court to amount to “double victimization” of the patient, with significant adverse consequences for ongoing treatment [56]. Undesired disclosure of patient records impairs treatment and can impact the procedural fairness of court proceedings that use clinical records as evidence [57]. Use of patient records as evidence can result in harm to the person’s psychiatric treatment.

Health professionals must consider privacy when providing health care through telehealth by taking reasonable steps to protect personal information from misuse and loss, and from unauthorized access, modification, or disclosure. The clinicians must advise on and obtain patient consent as to how their personal details will be collected, stored, and used [14].

#### Data Security

Data security has become a far more serious issue stemming from technology use in drug health clinics as greater volumes of data are generated and used in novel ways. Therefore, drug health information requires special protection against threats such as hacking and malware. Malicious or mistaken breaches of sensitive patient data can result in serious consequences such as reputational damage, emotional distress, stigma, and public health concerns [58]. Data security issues can also arise during telehealth consultations of people with SUD by being hacked when using video visits, using unprotected devices, public Wi-Fi, when there is low health digital literacy, or a lack of familiarity with online platforms [59]. Breach of data security during telehealth will have the same legal consequences as breaching data security of the patient’s medical record.

Recently, hacking by state actors has gained prominence. This is exemplified in the recent class action that is being brought against the private health insurer Medibank, where thousands of patient’s health records were published by ransomware hackers [58]. Frequently, hackers are external to health care and are linked to political or profit motives [58,60]. For example, the Australian National University lost the data of 200,000 patients covering a 19-year period due to a cyberattack believed to have been perpetrated by a sophisticated state actor [16]. In Australia, where those responsible for such attacks can be identified and brought before the courts, a range of criminal offenses exist under the Criminal Code Act 1995 (the Commonwealth; Cth) [61] and the Cybercrime Act 2001 (Cth) [45,62]. Australia participates in international approaches to support data security such as becoming a signatory to Europe’s Convention on Cybercrime [45,63]. However, it must be acknowledged that bringing perpetrators to justice is not always possible, and this does not undo the damage to patient confidentiality or privacy.

Thus, when providing health care through telehealth, patients need to be informed of privacy choices and data security measures the practitioner is adopting. Clinicians must avoid disclosure of sensitive personal information. In determining policy on data sharing, the interests of the individual need to be balanced against the risks [64].

#### Professional Indemnity and Liability

The legal ramifications regarding the provision of health care through telehealth consultation are largely untested. Courts will continue to consider what is reasonable in the provision of health care on a peer standard test. Practitioners who do not adopt appropriate technology or take reasonable and necessary precautions to secure patient information face potential legal action [65]. Clinicians, all users, software developers, and algorithm developers are all potentially liable. Hence, software and algorithm developers cannot be the only ones responsible for issues regarding technology [45].

Thus, health professionals should be aware that they have all the medicolegal risks of face-to-face consultations, and potentially an increased risk because of the limitations of telehealth in obtaining informed consent and the absence of physical examination. Special attention should be given to prevent errors and omissions, negligent credentialing, breaches of privacy, and interruptions of service due to equipment or technology failures. Clinicians need to be aware of what exactly their liability insurance policy covers, especially when providing telehealth services in other jurisdictions [47].

### Regulation of Telehealth Use in Australia

In Australia, technology-based patient consultation, including those for the drug health of patients, is regulated by the Health Practitioner Regulation National Law Act 2009 (NSW) [66], and the principles are defined in Good Medical Practice: a Code of Conduct for Doctors in Australia [67]. Privacy of communication is covered under the Telecommunications (Interception and Access) Act 1979 (Cth) [68], while confidentiality is regulated under The Privacy Act 1988 (Cth) [69]. Medical information handling for patients of drug health services is regulated under the Health Records and Information Privacy Act 2002 (NSW) [10]. Civil liability is determined by the principles of the Civil Liability Act 2002 (NSW) [70]. Finally, data security is regulated under the Criminal Code Act 1995 (Cth) [61], proposed by the Cybercrime Act 2001 (Cth) [62].

In other jurisdictions, for example in New Zealand, the confidentiality and privacy of patients with substance use issues have even stronger protections than in Australia. The New Zealand Evidence Act [71,72] states that strong privilege operates in criminal proceedings to protect communications between patients and health professionals when patients are being treated at drug health clinics for substance dependency under section 59 of the New Zealand Evidence Act. The rationale is that “the broader aim of securing due compliance with the law is more likely to be managed through medical treatment instead of through prosecution.” This particularly applies to drug addiction, where legal sanctions have minimum effect [72]. It is argued that New Zealand’s legislation better accommodates the needs of patients with drug addiction and mental health issues than its Australian counterpart for several reasons. First, it protects clinical records made when treating conditions that may lead to criminal behavior. Second, the legislation provides that the public interest in protecting vulnerable patients with drug addiction and mental issues is paramount. Finally, it seeks to protect the particular treatment relationship in question and ensures that potential patients are not prevented from seeking help. The New Zealand Evidence Act [71,73] was strengthened when the new Evidence Act was introduced in 2006 [74].

## Discussion

### Principal Findings

The findings from our review indicate that health care providers are required to consider 5 domains when using telehealth. The key precedent case laws are applicable when a breach of any of these 5 domains occurs. We found that telehealth use in patients with SUD and the arising legal considerations will remain an area of interest for medical and legal journals. This is not an unexpected outcome as telehealth can offer many benefits such as improving access to health care, decreasing medical costs, and increasing patient satisfaction [75].

Most published studies in the databases are in the English language. This is not surprising as English is the dominant language of international scientific publications; however, not all countries use it for medical research communications. Further, there is still a gap between the views of experts and researchers from other non–English-speaking countries [76]. It is worth noting that most published studies focused on using telehealth during the pandemic. The arguments and points about the legal considerations will likely continue and will identify future research areas.

Both medical and legal searches showed that telehealth use-related issues concern the 5 domains that were assessed with precedent case laws and legislation. A comparative legal analysis examining legal systems across different jurisdictions identifies similarities, differences, and best practices. The case laws related to autonomy, consent, confidentiality, and privacy are recognized in multiple countries including Australia, United States, United Kingdom, Canada, and New Zealand. This is related to sharing a common law tradition and similar legal systems. However, multiple other factors affect the recognition of foreign judgment including bilateral or multilateral treaties between countries, accessibility of legal sources in the foreign country, compliance with procedural requirements, public interest, and regulatory considerations. Such factors impact the enforceability and recognition of foreign judgments. In conclusion, recognizing foreign judgments is relevant because it provides cross-jurisdictional insights to support legal decision-making [77].

### Potentially Liable Parties From Telehealth Use

Our findings show that the following 3 parties can be legally liable for adverse clinical outcomes that arise due to the use of telehealth: the first is medical professionals and hospitals [45]. This liability depends on the nature of the breach such as whether the medical professional acted as part of good clinical practice as widely accepted by professional peer opinion [34]. The second is the software companies that provide the technology platforms [45]. This option focuses on whether the technology used provided acceptable specifications and protections. The third is that both parties can be liable [45]. This option is not blaming only one party as each party has a responsibility for the outcome.

### Regulation of Telehealth

Our study highlighted the regulatory perspective [45] as technology involves complex systems and has multiple medical, technology, and software specialties involved. Hence, it is difficult to regulate according to current law and policy. The regulation of medical devices is currently performed by the Therapeutic Goods Administration (TGA) [78]. The TGA states that Australian regulatory frameworks cannot be used to identify potential risk factors related to software such as in a medical device. Thus, the regulatory structure for the use of telehealth in drug health clinical settings is not established. The essential elements that must be considered in establishing the regulation of technology include (1) accessing the service, (2) controlling the operation of the service, and (3) identification and management of risks such as external hacking [79]. When new technologies are created there is often some initial concern from users [79], followed by assurances from service providers. When technological failure occurs, the law plays an important role in delivering redress, and through regulation preventing undesirable outcomes. Keeping regulatory frameworks up to date for every new technology is an ongoing journey [45].

Regulation of telehealth use and providing a quality standard from the TGA is difficult as there is no validated control procedure [45]. A collaborative approach comprising all relevant stakeholders including medical professionals, other specialists such as technology providers and insurance companies, is most likely to be successful in regulating telehealth use.

Our review suggested that a critical aspect is to avoid the occurrence of any harm and to ensure that the patient is fully informed of any potential risks or limitations of the proposed clinical service by telehealth [45]. Hence, health professionals conducting a consultation using telehealth in drug health situations should be aware that they may be legally liable in the event of misdiagnosis or treatment without consent that results in harm to the patient [80].

### Strengths and Limitations

This study has several strengths including the search of medical and legal basis principles, and the use of case law and statutes as primary legal sources. There are however several limitations to this study. First, this study included the English literature, and this limited comparison to studies published outside this domain and conducted in other countries. Second, this study included publications up to June 2022 as it sought to capture information before and during the COVID-19 pandemic.

### Conclusion

Our scoping review demonstrates that the implementation of telehealth in drug health settings is important to connect these patients to the required health care. Delivering health care through telehealth can be safe and effective if consent and autonomy, confidentiality, privacy, data security, and professional indemnity and liability are considered.

## Appendix

Multimedia Appendix 1 Detailed Search Strategy.

Multimedia Appendix 2 Preferred Reporting Items for Systematic reviews and Meta-Analyses extension for Scoping Reviews (PRISMA-ScR) Checklist.

## Abbreviations

Cth: the Commonwealth
NSW: New South Wales
PRISMA: Preferred Reporting Items for Systematic Reviews and Meta-Analyses
PRISMA-ScR: Preferred Reporting Items for Systematic Reviews and Meta-Analyses extension for Scoping Reviews
SUD: substance use disorder
TGA: Therapeutic Goods Administration
