# Supplementary material for: Telehealth Use and Legal Considerations in Drug Health Services During Pandemics: Systematic Scoping Review
Source: J Med Internet Res. 2024 Nov 12;26:e46394. doi: 10.2196/46394 (PMC11599891; doi:10.2196/46394)
Supplement: Multimedia Appendix 1 [file jmir_v26i1e46394_app1.docx]

**Method**

Since the review question involved legal and medical considerations, we decided that a systematic scoping review would be the most suitable. To achieve clarity and accuracy and to avoid poor reporting we applied Preferred Reporting Items for Systematic Reviews and Meta-Analysis extension for Scoping Reviews (PRISMA-Scr)^1^

**Review context**

What is known from legal and medical literature about use of telehealth, current regulations related to telehealth use in drug health services, relevant case laws, international regulatory models, and what are the legal considerations using telehealth in drug health service?

**Inclusion Criteria**

Inclusion criteria

• Those related to telehealth use

• Those related to regulations and legal considerations when using telehealth

• All types of publications were included: original studies reviews and editorials, viewpoints, guidelines, letter to editors and commentaries,

• The articles should have been published in a peer reviewed platform or be an institutional report

• English reports were reviewed.

**Exclusion criteria**

• Those irrelevant to telehealth use

• Those related to telehealth modelling, and studies that used technology only for a better understanding of disease dynamics with no help for health professionals

• Preprints or unpublished data

• Unavailable full texts, or full name of sources.

**Search strategy**

We performed a medical and a legal research.

**Medical research**

First, a limited preliminary search was performed using multiple databases including Google Scholar and PubMed for identifying the appropriate keywords. Second, in June 2022 we established a search strategy using electronic databases such as Cochrane Library, PsycINFO, Pub- Med, Cinahl, Embase, Scopus, and the Web of Sciences. Other relevant websites were explored including those of the Global Health Library, WHO, United States Food and Drug Administration, European Medicines Agency, and Australian Government Department of Health. Other search engines such as Google Scholar, ResearchGate and Science Direct were explored using the search terms. The results were checked by using Endnote 20. After duplicate publications were eliminated, the authors checked the titles and abstracts and eliminated irrelevant studies based on the inclusion and exclusion criteria. Discrepancies regarding the inclusion/exclusion of any report were decided through discussion. Third, the authors checked the included papers’ reference lists for identifying directly relevant context. In the fourth step, we checked google scholar for publications’ citations and current publications, maximizing our research to obtain all relevant reports. Any discordance was discussed for final selection in the third and fourth steps. We performed the literature search using the following keywords: telehealth use; emergency department admission; drug addiction; consenting; confidentiality; data safety.

**Legal research**

Extensive legal research was undertaken using Australian Case Law databases including Westlaw AU, Lexis Advance Pacific, AustLII, CCH Intelliconnect, and CaseLaw NSW. The relevant legislation in other countries was determined. The key terms including autonomy consent, confidentiality, privacy, data security, professional indemnity, liability, Privacy Act, legal basis for telehealth were used for the search.

**Report selection and Data extraction**

Articles selected by our search strategy were imported onto EndNote and duplicates were removed^2^. MJ reviewed the title and abstracts of the identified articles. All authors checked the included studies and was able to confirm that the inclusion criteria were correctly applied. We created a Microsoft Excel sheet for data extraction to list specific data from the included articles, including journal title, reference to telehealth or its variants in the article title, reference to legal considerations or its variants in the article title, publication type, focus of the article, terminologies used to describe telehealth and their accompanying definitions, the aim of telehealth use, and the drug health settings or medico legal specialty and legal considerations. Moreover, for the original research, we included the following data from the publications: methodological approach, critical analysis, policy, case law and regulatory related research. All publications were reviewed by all authors. One of the importance of this study is authors are medical and legal background and able to critically analyse medical and legal part of the review.

**Results**

We retrieved 300 records from our medical search and 340 records from our legal search. Then 496 records were screened for relevance of title and abstract, excluding 260 of the records 236 full records were reviewed for eligibility. 156 of them were excluded as they were not directly relevant to the context of the current study. Finally, 80 studies (43 legal and 37 medical studies) were included. The bibliography of all selected articles was also reviewed for relevance.

**Table 1 Keywords and search strategy**

**Database Search strategy and keywords for medical search**

PubMed **Title, abstract and keywords** telehealth use; emergency department admission;

drug addiction; consenting; confidentiality; data

safety.

Cochrane Library

PsycINFO

Cinahl,

Embase

Scopus

**Database Search strategy and keywords for legal search**

Westlaw AU **Title, abstract and keywords** autonomy consent, confidentiality, privacy, data

security, professional indemnity, liability, legal basis for telehealth were used for the search.

Lexis Advance Pacific

AustLII

CCH Intelliconnect

**Types of included papers**

The distribution of the publications, regarding the types of publications, is provided in Figure 1. Of the 80 publications (Table 2), 37 (46.2%) presented medical research as follows: Eleven original articles [1,7,11,13-14,17-18,26,43,73,76], two review [41,55], five scoping review [5,6, 49,57,75], eight commentaries [9,15-16,22,44,66,72,74], six special articles [10,21,27,45,50,63], and five viewpoints [12,23,24,28,46]. 43 (53.7%) presented as legal research as follows: Three books [2,79,80], four legal literature [19,31,52,54], five legal reviews [3,4,30,64,77], fifteen Acts [8,25,34,35,37,39,40,60,61,65,67,68,69,70,71], three case laws [29, 32,51], two critical analyses [33,36], four legal commentaries [20,47,48,56], two news [38,78], one policy brief [53], and four perspectives [42,58,59,62].

**Figure 1 Distribution of included papers based on types of publications**

**Table 2 The distribution of presentations, based on types of publications**

**Type of publication Total number**

**Medical Research [1,5-7,9-18,21-24,26-28,41,43-46,49-50,55,57,63,66,72-76] 37**

Original articles [1,7,11,13-14,17-18,26,43,73,76] 11

Review [41,55] 2

Scoping Review [5,6, 49,57,75] 5

Commentary [9,15-16,22,44,66,72,74] 8

Special Article [10,21,27,45,50,63] 6

Viewpoint [12,23,24,28,46] 5

**Legal Research[2-4,8,19-20,25,29-40,42,47-48,51-54,56,58-62, 64-65,67-71,77-80] 43**

Book [2,79,80] 3

Legal Literature [19,31,52,54] 4

Legal Review [3,4,30,64,77] 5

Act [8,25,34,35,37,39,40,60,61,65,67,68,69,70,71] 15

Case Law [29, 32,51] 3

Critical Analysis [33,36] 2

Legal Commentary [20,47,48,56] 4

News [38,78] 2

Policy Brief [53] 1

Perspective [42,58,59,62] 4

**Categories of included papers**

The legal and medical issue was identified by presenting the original article [1-4], research methods followed by scoping reviews [5,6], why telehealth is needed at drug health services and drug health patient characteristics presented by original publications [7-10,12-13], commentary [11, 15, 17-18], review[14], and legal review [19]. The legal considerations were categorised into five elements. The firstly autonomy and consent is presented by reviews [20, 26] original publications [21,23], commentaries [22, 24-25], legal reviews [27-28, 30], acts [29], legal commentaries [31], systematic reviews [32], reports [33], special articles [34-35]. The second, confidentiality is presented by reviewing original articles [36,39,40], books [37], case laws [38], legal analysis [41,42], viewpoints [43]. Thirdly privacy is presented by reviewing original articles [36,39,40], books [37, case laws [38], legal analysis [41,42], viewpoints [43]. Fourthly, data security is presented by systemic reviews [32], news[44], policy briefs [45], perspectives [46], commentaries [47]. Fifthly professional indemnity and liability is presented by reviewing legal commentary [48], original articles [49-51], commentaries [52], reviews [53], books [54], and critical analysis [55]. Then current regulation in Australia is presented by reviewing original studies [49], compared with other countries by original study [50-51]. We also discuss benefits of telehealth by reviewing legal reviews [27] and systematic reviews [32]. Regulatory perspective is presented by analysing systematic reviews [32], reviews [53] , and books [54].

Significant points of this paper are firstly that to the best of our knowledge, this is the first scoping review showing the benefits of the use of telehealth with comprehensive medical and legal considerations. Secondly, this research found that legal considerations are critical when using telehealth for patients, health professionals, and health service providers. Finally, the study team included medical and legal professionals and each statement was assessed critically.

1. Tricco AC, Lillie E, Zarin W, et al. PRISMA extension for scoping reviews (PRISMA-ScR): checklist and explanation. *Annals of internal medicine.* 2018;169(7):467-473.

2. Peters MD. Managing and coding references for systematic reviews and scoping reviews in EndNote. *Medical Reference Services Quarterly.* 2017;36(1):19-31.
